# An Enhanced Social Networking Intervention for Young People with Active Suicidal Ideation: Safety, Feasibility and Acceptability Outcomes

**DOI:** 10.3390/ijerph17072435

**Published:** 2020-04-03

**Authors:** Eleanor Bailey, Mario Alvarez-Jimenez, Jo Robinson, Simon D’Alfonso, Maja Nedeljkovic, Christopher G. Davey, Sarah Bendall, Tamsyn Gilbertson, Jessica Phillips, Lisa Bloom, Laura Nicholls, Nicola Garland, Daniela Cagliarini, Mark Phelan, Ben McKechnie, Jessica Mitchell, Melanie Cooke, Simon M. Rice

**Affiliations:** 1 Orygen, Parkville, Victoria 3052, Australia; eleanor.bailey@orygen.org.au (E.B.); Mario.alvarez@orygen.org.au (M.A.-J.); jo.robinson@orygen.org.au (J.R.); dalfonso@unimelb.edu.au (S.D.); christopher.davey@orygen.org.au (C.G.D.); sarah.bendall@orygen.org.au (S.B.); tamsyn.gilbertson@orygen.org.au (T.G.); jess.phillips@orygen.org.au (J.P.); lisa.bloom@mh.org.au (L.B.); laura.nicholls@mh.org.au (L.N.); nicola.garland@mh.org.au (N.G.); daniela.cagliarini@orygen.org.au (D.C.); mark.phelan@orygen.org.au (M.P.); ben.mckechnie@mh.org.au (B.M.); jessica.mitchell@mh.org.au (J.M.); melanie.cooke@orygen.org.au (M.C.); 2 Centre for Youth Mental Health, University of Melbourne, Parkville, Victoria 3010, Australia; 3 Swinburne University of Technology, Hawthorn, Victoria 3122, Australia; mnedeljkovic@swin.edu.au; 4 School of Computing and Information Systems, University of Melbourne, Parkville, Victoria 3052, Australia

**Keywords:** suicide, young people, internet, social media, Interpersonal Theory of Suicide, interventions

## Abstract

Online social networking interventions have potential to support young people who experience suicidal thoughts by specifically addressing interpersonal risk factors for suicide, but may also pose a risk of harm. This uncontrolled, single-group pilot study aimed to evaluate the safety, feasibility, and acceptability of an enhanced online social networking intervention (“Affinity”) among a sample of young people who experienced active suicidal ideation, and to explore potential changes in clinical outcomes and the therapeutic targets of the intervention. Twenty young people with current or recent suicidal ideation who were receiving treatment for depression at a tertiary-level mental health service were given access to Affinity for two months. Participants were assessed at baseline and 8-week follow-up; 90 percent reported clinical suicidal ideation at baseline. A priori criteria related to feasibility, safety and acceptability were satisfied. In terms of potential clinical effects, significant and reliable pre-post improvements were found on self-report outcomes including suicidal ideation. This study provides initial world-first evidence to support the use of an online intervention incorporating social networking as an adjunct to treatment for young people who experience suicidal ideation. The effectiveness of Affinity needs to be evaluated in a randomised controlled trial.

## 1. Introduction

Suicide is a leading cause of mortality in young people worldwide, and the rate of youth suicide is increasing in many countries including Australia and the USA [1,2,3,4]. The World Health Organization considers suicide a major public health issue and has emphasised the importance of targeting new suicide prevention approaches to individuals and groups who are at particular risk of suicide [5,6]. Given that depressive disorders [7] and history of suicidal ideation and behaviour [8] are among the strongest predictors of future suicidal behaviour, there is a need for targeted suicide prevention approaches to support young people exhibiting these risk factors.

Although young people with depressive disorders and suicidal ideation frequently receive treatment from traditional mental health services (typically involving individual face-to-face sessions with a trained professional), these may be limited in several ways. First, suicide risk fluctuates frequently and unpredictably [9], yet services are typically unable to provide ongoing or real-time support to clients between sessions. Second, funding models and demand for services often restrict the number of sessions clients can access, which can result in clients’ premature discharge without adequate supports in place; this is particularly concerning given the risk of suicide is heightened following discharge from mental health services [10]. Third, young people who experience suicidal thoughts and behaviours may not access mental health services at all due to practical or stigma-related barriers [11]. Finally, mental health services may be limited in their capacity to target interpersonal risk factors for suicide, such as the constructs of perceived burdensomeness and thwarted belongingness referred to in Joiner’s Interpersonal Theory of Suicide (IPTS) [12,13].

Internet-based interventions may have the capacity to overcome some of the limitations of traditional mental health treatment, in that they can be available 24 h a day at low-to-no cost and without geographical constraints, and they can be accessed from the privacy of home [14]. Where internet interventions facilitate mutual support between peers, they may also be able to target interpersonal risk factors for suicide: involvement in an online community of similar others who openly and supportively discuss their experiences and challenges with mental health may foster a sense of belongingness, whilst the opportunity to provide online support to others may lead to reductions in perceived burdensomeness [15]. Internet-based interventions that deliver evidence-based therapeutic content, such as websites based on cognitive behavioural therapy, have shown promise for people who experience suicidal thoughts and behaviours [16,17,18]. To date, however, one of the key benefits of the internet for suicide prevention – the facilitation of mutual support between peers [14,19] – has not been harnessed in the implementation of an intervention to support people at high risk of suicide [20]. Internet-based interventions that facilitate peer-to-peer support may be particularly appealing to young people, who use the internet and social media on a daily basis [21]. Finally, although several stand-alone interventions have been developed and tested, digital interventions are rarely, if ever, integrated into clinical care for this population. Integration of digital interventions with clinical care may enhance the accessibility, continuity and intensity of treatment, addressing the limitations of currents models of care [22], and is therefore worthy of exploration.

Despite the potential of internet-based social networking interventions for supporting youth at risk of suicide, there is concern about possible risks such as normalising suicidal behaviours and reinforcing them [15,23]. Additionally, just as clustering and contagion of suicidal behaviour have been demonstrated to occur in offline social networks [24,25], there may be a risk of such phenomena occurring in online settings, perhaps to an even greater degree [26,27]. Therefore, although online interventions incorporating theory-driven therapeutic content and peer-to-peer support may have great potential to target risk factors related to suicide in a format that is engaging and accessible to youth, they must incorporate considered safety procedures, including moderation and regular monitoring of user-generated content.

In seeking to enhance the benefits afforded by digital technologies, whilst also mitigating the risks, eOrygen, the digital mental health division of Orygen, developed the Moderated Online Social Therapy (MOST) model [28,29]. The MOST model incorporates three main components: online social networking, expert and peer moderation, and therapeutic content delivered in various formats including graphic medicine comics. Successive iterations of the MOST model have been adapted for, and evaluated with, young people with psychosis [30], and at high risk of developing psychosis [31], young people with depression [32], and social anxiety [33], help-seeking young people [34], and carers of young people with psychosis [35]. To date, however, those at high risk of suicide have been excluded from participating in these studies, and are also typically excluded from other studies of online interventions for mental health problems [36]. Given the potential the MOST model has to support people who experience suicidal thoughts, and that it has been found to be safe for young people experiencing serious mental health problems [28,32], adaption of the model for a population of young people who experience suicidal ideation is an obvious next step.

The aim of the current study was to evaluate the safety, feasibility, and acceptability of a MOST intervention (“Affinity”) among a sample of young people who were receiving treatment for major depressive disorder and had also experienced past-four-week suicidal ideation. A secondary aim was to explore changes in cognitive and interpersonal targets of the Affinity intervention, as well as changes in self-reported depression and suicidal ideation.

## 2. Methods

### 2.1. Design

This study employed an uncontrolled single-group pre-test post-test design with assessments administered at two time-points (baseline and 8-week follow-up).

### 2.2. Participants

Participants were current clients of the Youth Mood Clinic (YMC), a tertiary-level outpatient mental health service that is part of Orygen, a state government-funded youth mental health service in Melbourne, Australia. YMC specialises in the treatment of young people with complex depression; that is, young people with major depressive disorder or non-psychotic bipolar disorder plus two or more of: suicide risk; comorbid mental disorders; poor psychosocial functioning [37]. Inclusion criteria were: 1) aged 16 to 25 years inclusive; 2) suicidal ideation within the past four weeks, as determined by a single screening question; 3) engagement with treatment (e.g., regular attendance at appointments; not approaching discharge from the service); 4) familiarity with, and willingness to use, available crisis supports; 5) ability to give informed consent and comply with study procedures; 6) regular and ongoing internet and telephone access; and 7) willingness to nominate at least two emergency contacts. There were no specific exclusion criteria related to level of suicide risk. Clinicians were consulted on a case-by-case basis regarding participant suitability.

### 2.3. Procedure

Participants were recruited over a five-month period from April to August 2018, and were referred to the study by their treating clinicians at the YMC. Interested participants met with the lead author to obtain more information about the study, and, as appropriate, provide informed consent and complete a brief screening interview, and baseline assessment. and receive an orientation to the Affinity website and provided with log-in details immediately following the baseline assessment, and there were no Affinity users other than members of the study team and consenting participants; in other words, the number of Affinity users increased over the recruitment period. Participants were informed that they could access Affinity at their convenience, as much or as little as they liked, until the website was closed (October 31st, 2018). Approximately eight weeks after their baseline assessment, each participant was invited to meet with the lead author again to complete the follow-up assessment. Participants were reimbursed AUD$60 for each assessment.

### 2.4. Intervention

Affinity is an interactive, purpose-built online platform which was designed as a supplement to traditional face-to-face interventions for young people with suicidal ideation. The platform was established specifically for the purpose of this research project and was live from March to October 2018. Affinity follows the MOST model. Each element of Affinity is described below.

#### 2.4.1. Peer-to-Peer Online Social Networking

Affinity includes a newsfeed, called the “Café”, where participants and moderators can post comments, information, upload pictures and videos, and reply to content posted by other users.

Users can also “like” and “react” to different content; react responses are predefined and include “thinking of you”, “feeling the same” and “LOL”. Although the MOST software has capability for a real-time private chat function between users, this function was disabled for this study. Users could post on each other’s profile pages, but all communication between users was visible to the wider Affinity network. Although users are not permitted to include swearwords in regular posts, Affinity includes a “vent post” function. If “I’m just venting” is selected, the system covers the post with a statement warning users of possible inflammatory content (e.g., swearwords) and requiring them to elect to view it via clicking a button.

Affinity also incorporates an online problem solving forum, called “Talk It Out”, in which users can nominate issues they are facing. Once a suggestion is approved and made public by the peer moderators, other users are invited to contribute possible solutions. Talk It Out is based on an evidence-based problem solving framework [38,39] and sequentially follows four phases: 1) defining the problem; 2) brainstorming solutions; 3) discussing the pros and cons to each solution; 4) summarising the discussion.

It is anticipated that by interacting and exchanging mutual support with others using the social networking function of Affinity, users may experience improvements in social connectedness and in the interpersonal constructs of the IPTS [15].

#### 2.4.2. Moderation

Affinity incorporates two types of moderation: clinical moderation and peer-moderation.

Clinical Moderation: Affinity was moderated by two expert youth mental health clinicians from the Youth Mood Clinic, who each allocated the equivalent of one full day per week to this role, typically spread over several days. The clinical moderator role was to provide guidance, prompt participation in discussions within the Affinity network, monitor participants’ wellbeing and ensure the safety of the social networking environment. On entry to the study, each Affinity user was assigned to a particular moderator, who was then primarily responsible for monitoring that participant’s Affinity activity, and engaging them for the duration of the trial. Because the clinical moderators were integrated within the clinical service from which participants were recruited, communication about risk or wellbeing issues between Affinity staff and the participants’ treating clinicians was timely and accurate. This also meant that the clinical moderators had a comprehensive understanding at the outset of the general issues experienced by Affinity users, as well as their current treatment circumstances.

Peer Moderation: The role of peer moderators was to provide guidance, information and emotional support to Affinity users. These were young people who had been discharged from the Orygen Youth Health clinical program and had trained to become Peer Support Workers. The peer moderators managed the Café and Talk It Out sections of the system, with support from the clinical moderators and wider research team as needed. Several measures were in place to ensure the mental wellbeing of the peer moderators, including 1) the provision of comprehensive training prior to using Affinity, including training regarding the limitations of their role and emphasis that they were not expected to manage clinical risk; 2) fortnightly supervision with a dedicated Peer Worker Coordinator, plus additional supervision as required; 3) monthly supervision with senior clinicians from the research team.

#### 2.4.3. Therapeutic Content

Rather than a static text-based approach, therapeutic content on Affinity is delivered via purpose-developed comics, called “Steps”. There are 58 individual Steps on Affinity, and each is approximately 15-25 panels in length. Steps are grouped on Affinity according to the following general areas: communication, mindfulness, self-compassion, depression, anxiety, social anxiety, and strengths. These general areas are called “Pathways”. Affinity users can choose to progress sequentially through the Steps using Pathways, or access them individually and at random. The general Pathways and content of the Steps is based on literature regarding what works in treating youth depression together with the therapeutic targets described in the IPTS [12,13]. This is described in detail elsewhere [15]; for example, however, Steps targeting communication skills and the identification and promotion of strengths may subsequently reduce perceived burdensomeness or increase ability to connect with others, CBT-based Steps on depression and anxiety illustrate the role of thoughts, emotions and behaviour in maintaining and addressing negative mood states, and Steps based on mindfulness approaches aim to reduce symptoms of depression and hopelessness [15].

Steps were developed iteratively, through a structured multi-stage process of youth consultation, scripting, illustration and inter-disciplinary feedback. Each step suggests a number of actions (“Do Its”) users can take to put the information into practice, such as listening to a mindfulness audio track, identifying emotions from a list, and creating a wellness plan. Users are able to save Do Its to a playlist and revisit them at a later date, and are encouraged to mark them complete after they have done them.

#### 2.4.4. Safety and Security Measures

Several safety and security measures were put in place to minimise the risk of harm on Affinity. First, participants were required to agree to a “terms of use” prior to accessing Affinity. In addition to outlining the types of posts that would be considered unacceptable (e.g., bullying or harassment) and emphasizing the intermittent nature of the moderation and consequent inability of the moderators to respond in an emergency, the terms of use required participants not to share their personal contact information with other Affinity users. The terms of use also explained to participants that any concerns about their wellbeing based on information passed onto moderators or communicated within the system may be communicated with their treating clinician.

Second, in addition to the clinical moderation, Affinity “safety checks” were performed twice daily on weekdays and once daily on weekends by a member of the research team. When completing a safety check, the team member reviewed new content posted by participants since the previous safety check for any content indicating possible risk. In addition, an automatic keyword detection system detected and blocked any posts containing key words that may indicate suicide risk for the user who posted the comment. In the event that this system was activated, an automated text message was sent to a nominated member of the research team; this phone was accessed during business hours.

The safety protocol designed for this study required that, in any instance where there was concern for a participant’s wellbeing or safety, the relevant moderator or research team member would attempt to contact the participant by phone to complete a risk assessment. If the participant could not be contacted by phone, their first and second emergency contacts would be phoned sequentially as necessary. If neither emergency contact could be reached, and there was significant concern for the participant’s safety based on the nature of their post, emergency services would be called. In all cases where the safety protocol was activated, the participant’s treating clinician was informed and, where possible, asked to follow up with the participant and manage the risk.

Additionally, a range of measures were in place to help ensure the security of the website and the data collected by it. Affinity was hosted on a secure university web server, which has standard measures in place to prevent unauthorised access. In addition, the web application includes measures to secure the application and database against unauthorised access. These measures conform to industry best practice as defined by the Synergy guidelines [40] and the Open Web Application Security Project (www.owasp.org).

### 2.5. Outcomes

#### 2.5.1. Safety, Feasibility and Acceptability

A priori criteria were set regarding safety, feasibility and acceptability. Safety criteria were: 1) no adverse events (e.g., suicide attempt) attributed to the Affinity intervention; 2) no overall deterioration on measures of self-reported depression or suicidal ideation; 3) average score of 4 or more obtained on the safety item in the quantitative measure of subjective experience of Affinity, where a score of 5 corresponds to “very safe”. All adverse events and serious adverse events were clearly documented, tracked and reported on, and a determination about the potential causative role of the Affinity system in any of these events was made by the study medical officer. Subjective experience of Affinity was evaluated using a quantitative questionnaire at follow-up. This measure contained eight items relating to participants’ experience of Affinity in general, as well as up to 16 items relating to its specific components. Each item was rated on a scale from 1 to 5, where 5 corresponded to the most positive rating. Specific anchors varied between items; see Appendix 1 for the full measure.

Criteria related to feasibility were based on recruitment rates in previous studies using the MOST platform and were: 1) recruitment target (n = 20) reached; 2) less than 50 percent of young people approached by the research team about Affinity refused participation. Criteria related to acceptability were: 1) at least 50 percent of participants logged on at least once per week for the duration of the intervention period; 2) participants provided positive feedback (i.e., mean score of 3 or higher on key items) using the purpose-designed measure of subjective experience on Affinity. Frequency, duration and patterns of use were tracked in real time by the Affinity system.

#### 2.5.2. Self-Report Outcomes

In addition to a standard demographic questionnaire at baseline, participants completed a battery of self-report measures at baseline and follow-up relating to clinical outcomes (suicidal ideation and depression), interpersonal targets related to the IPTS (perceived burdensomeness, thwarted belongingness, and social connectedness) and cognitive targets of the intervention (mindfulness, self-compassion, and problem solving); these are described in detail below.

Suicidal ideation was assessed using the Adult Suicidal Ideation Questionnaire (ASIQ) [41], a 25-item instrument designed to assess the frequency of occurrence of cognitions associated with suicidal ideation among adults. Each ASIQ item is scored on a Likert-type scale from 0 to 6. Total scores range from 0 to 150, with higher scores indicating greater degree of suicidal ideation; scores of 31 or higher indicate need for further evaluation of suicidal behaviour [40]. Internal consistency in the sample was excellent (α =0.96 at baseline; α = 0.98 at follow-up).

Depression was measured using the Patient Health Questionnaire-9 (PHQ-9) [42], a 9-item measure that requires participants to indicate how often they have been bothered by nine problems over the past two weeks. Each item is rated on a four-point Likert scale ranging from 0 (not at all) to 3 (nearly every day), with higher scores indicating more severe depressive symptoms. Internal consistency in the sample was acceptable at baseline (α = 0.71) and excellent at follow-up (α = 0.90).

Perceived burdensomeness and thwarted belongingness were measured using the 15-item version of the Interpersonal Needs Questionnaire (INQ-15) [43]. Each item is scored on a Likert-type scale from 1 to 7, with higher scores reflecting greater thwarted belongingness and perceived burdensomeness. The INQ-15 has demonstrated good psychometric properties, and internal consistency in the sample was good at baseline (α = 0.86) and excellent at follow-up (α = 0.93).

Social connectedness was measured using the Social Connectedness Scale-Revised (SCS-R) [44]. Participants respond to eight items using a 6-point Likert scale ranging from 1 (Strongly Agree) to 6 (Strongly Disagree). Higher scores reflect a greater sense of connection to society. Internal consistency in the sample was excellent (α = 0.92 at baseline; α = 0.97 at follow-up).

Mindfulness was measured using the Mindful Attention Awareness Scale (MAAS) [45] a 15-item measure of dispositional mindfulness (open or receptive awareness of and attention to what is taking place in the present). Items are rated on a 6-point Likert scale from 1 (almost always) to 6 (almost never). The scale is scored by computing a mean of the 15 items, with higher scores reflecting higher levels of dispositional mindfulness. The scale shows strong psychometric properties. Internal consistency in the sample was acceptable at baseline (α = 0.75) and good at follow-up (α = 0.85).

Self-compassion was measured using the Self-Compassion Scale – Short Form (SCS-SF) [46]. Twelve items are rated from 1 (almost never) to 5 (almost always) with the total score derived by adding the means of each subscale together; higher scores reflect greater self-compassion. Internal consistency in the sample was good (α = 0.84 at baseline; α = 0.83 at follow-up).

Problem solving was assessed using the Negative Problem Orientation Questionnaire (NPOQ) [47]. The NPOQ contains 12 items measure assessing negative problem orientation, defined as a dysfunctional cognitive set that comprises the tendency to see problems as a threat, doubt one’s own problem-solving ability, and be pessimistic about the outcome [47]. Participants rate each item using a 5-point Likert scale ranging from 1 (not at all true of me) to 5 (extremely true of me). Items include “I see problems as a threat to my well-being” and “I often see problems as bigger than they really are”. Item scores are summed to arrive at a total score, with higher scores indicating more dysfunctional problem solving attitudes. Internal consistency in the sample was good at baseline (α = 0.86) and excellent at follow-up (α = 0.93).

Additionally, incidence of suicide attempt in participants’ lifetime (time 1) and since baseline (time 2) was assessed using purpose-designed items based on the Columbia-Suicide Severity Rating Scale (CSSRS) [48], and incidence of self-harm was assessed using the Deliberate Self-Harm Inventory (DSHI) [49]. The DSHI assesses the incidence of 16 different self-harm behaviours, as well as any other self-harm behaviour not asked about.

### 2.6. Ethics

The study received approval from the Melbourne Health Human Research Ethics Committee (ID 2017.187). All participants provided informed consent prior to participating. Participants under the age of 18 were required to also provide consent from a parent or guardian.

### 2.7. Statistical Analysis

Frequency, duration and patterns of use were tracked in real time and analysed using descriptive statistics. Within-subject analyses (paired samples t-tests) were conducted and effect sizes (Cohen’s d) reported for change to continuous variables between baseline and 8-weeks. Morris and DeShon’s equation [50] was used to estimate Cohen’s d, as this corrects for dependence among means.

The Reliable Change Index (RCI), computed by dividing the difference between the pre-treatment and post-treatment scores by the standard error of the difference, was used to determine whether change in individual scores was reliable and statistically significant [51]. A RCI of 1.645 or higher indicates that the individual change score is significantly greater than it would be expected due to random measurement error [52,53]. Due to the skewed distribution of the variables, associations between usage of Affinity and changes in clinical outcomes were explored using non-parametric correlations (Kendall’s tau-b).

## 3. Results

### 3.1. Participants

Forty-six YMC clients were identified by treating clinicians as meeting inclusion criteria and were referred to the study. Of these, 23 accepted (50%), 10 declined (23%), and 13 could not be contacted (28%). Reasons for declining were general lack of interest (n = 6), or disliking social media and/or socialising with others (n = 4). Of those who accepted, three were subsequently deemed ineligible at baseline because they had not experienced suicidal ideation within the past four weeks. Ultimately 20 participants were successfully recruited to the study.

Of the 20 participants recruited, two (10%) were lost to follow-up as they did not respond to texts or calls from the research team; 90% completed the eight-week follow-up assessment. Demographic and clinical characteristics are reported in Table 1 and Table 2. Just over half the participants were female (n = 11; 55%), two (10%) identified as transgender, and seven (35%) were male. The mean age at baseline was 21.7 years (SD = 2.7). Sixteen (80%) reported their marital status as “single”. Regarding sexual orientation, 12 (60%) identified as heterosexual, one (5%) as gay, and three each (15%) as either bisexual or questioning. Fifteen (75%) were born in Australia, and no participants identified as Aboriginal or Torres Strait Islander. Fourteen (70%) were currently studying or training and 16 (80%) were currently employed.

At baseline, on the PHQ-9, 85% (n = 17) of the sample scored in the “moderately severe” (PHQ-9 = 15-19) or “severe” (PHQ-9 = 20+) range for depression, and 90% (n = 18) scored in the clinical range (≥31) on the ASIQ. Thirteen participants (65%) had been hospitalised due to either severe suicidal ideation or suicide attempt in their lifetime, with 11 (55%) reporting a previous suicide attempt and eight (40%) reporting planning for suicide (e.g., by writing letters to family or collecting medications for overdose). Twelve participants (60%) reported attempting or planning for suicide within the past six months. Sixteen (80%) endorsed at least one self-harm behaviour in their lifetime, with 15 (75%) engaging in two or more, 10 (50%) in three or more, and six participants (30%) endorsing five or more self-harm behaviours. The most common self-harm behaviour endorsed by participants was cutting (81% of those who had self-harmed reported using this method).

### 3.2. Safety

Two serious adverse events (suicide attempts requiring hospitalisation) were reported during the intervention period. Hospitalisation for suicide risk is not uncommon for young people attending YMC, and in both cases the study doctor (an experienced consultant psychiatrist) determined that these events were not related to the Affinity intervention. Moreover, one of these participants used the system more than average (11 weekly log-ins and 41 posts/comments on the newsfeed) and one less than average (2 weekly log-ins and 3 posts/comments on the newsfeed), suggesting a relationship between frequency of system usage and incidence of suicide attempt was unlikely. There was no overall deterioration on measures of depression or suicidal ideation, with only two participants showing a reliable decline on these outcomes (Table 3). Finally, a mean score of 4.8 (SD 0.56) from a maximum of 5 was obtained on the item related to safety in the subjective experience questionnaire (Table 4).

### 3.3. Feasibility and Acceptability

As reported above, the recruitment target of 20 participants was met, with only 23% refusing participation. Affinity usage data is displayed in Table 5. Despite significant variability across participants, overall more than half the participants logged in at least once per week satisfying this criterion related to acceptability. There was also significant variability in Café activity (including posts, replies, and likes/reactions), Steps and actions completed, and amount of user-initiated contact with moderators.

Participants provided positive ratings of their experience with Affinity (see Table 4), with mean scores of 3 or more achieved on each of the core items. The mean ratings indicate that participants perceived Affinity to be helpful, easy to use, safe, and a positive experience, with 100% of participants rating these items positively (3 or higher). Mean scores of 3.8 and 3.2 indicate Affinity helped participants to feel more in control of their mood and suicidal thoughts, with 94% and 81% of participants respectively providing positive ratings on these items. Finally, 16 participants (90%) reported they would use Affinity outside the research project, and 18 (100%) said that they would recommend Affinity to other young people who experience suicidal thoughts.

### 3.4. Potential Clinical Effectiveness

Examination of the self-report outcomes indicate significant improvements with a medium size effect, between baseline to follow-up in suicidal ideation (*d* = −0.57, *p* = 0.033) and perceived burdensomeness (*d* = −0.52, *p* =.048), with 41% and 39% of participants demonstrating reliable improvements in these variables respectively. There were significant large improvements in depressive symptoms (*d* = −0.94, *p* = 0.016), thwarted belongingness (*d* = −0.96, *p* = 0.006), and self-compassion (*d* = 0.82, *p* =.004). Forty-one percent of participants demonstrated reliable improvements on depressive symptoms, 56% did so on thwarted belongingness and self-compassion. There were no significant differences from baseline to follow-up in problem-solving, mindfulness or social connectedness; however, 44% demonstrated reliable improvements on problem solving and social connectedness (see Table 3).

### 3.5. Exploratory Correlations Between Affinity Use, Subjective Ratings and Outcomes

Exploratory non-parametric correlations revealed several medium-to-large significant correlations between elements of Affinity use and both favourable subjective ratings of Affinity and improvements in outcomes (Table 6). For example, large correlations were found between number of Steps completed and both reduced depression symptoms (*rt* = −0.61; *p* < 0.01) and perceived burdensomeness (*rt* = −0.60; *p* < 0.01). Medium positive correlations were found between higher ratings of perceived general helpfulness and Café posts/comments (*rt* = 0.47; *p* < 0.05), Steps completed (*rt* = 0.39; *p* < 0.05), and Do-Its completed (*rt* = 0.46; *p* < 0.05). Large positive correlations were found between rating Affinity as helping to feel more in control of mood and: likes/reactions made (*rt* = 0.60; *p* < 0.01); likes/reactions received (*rt* = 0.54; *p* < 0.01); Café posts/comments (*rt* = 0.56; *p* < 0.01); and Do-Its completed (*rt* = 0.53; *p* < 0.01). A medium positive correlation was found between positive ratings of Affinity’s ability to help control mood and total number of logins (*rt* = 0.45; *p* < 0.05). Please refer to Table 6 for all significant correlations identified.

## 4. Discussion

This is the first study to pilot test a theoretically and empirically-driven online intervention incorporating peer-to-peer social networking [15] with a population of young people who experience suicidal ideation. Results support the safety of Affinity: it was perceived to be safe, no adverse events occurred that were attributed to the intervention, and no overall deteriorations were found on self-reported depression or suicidal ideation (nor any other clinical or social measure). The recruitment target was met with less than one-quarter of those approached refusing participation, indicating feasibility. Finally, participants perceived Affinity as easy to use, helpful, and a positive experience, and logged on at least once per week on average during the trial, supporting the acceptability of Affinity. Providing further support for both feasibility and acceptability, only two participants were lost to follow-up. Exploratory examination of clinical outcomes, and cognitive and interpersonal therapeutic targets of the intervention, revealed significant large reliable improvements on depressive symptoms (*d* = −0.94, 41% of participants reliably improved), thwarted belongingness (*d* = −0.96, 56% improved), and self-compassion (*d* = 0.82, 56% improved) and medium significant reliable improvements on suicidal ideation (*d* = −0.57, 41% improved) and perceived burdensomeness (*d* = −0.52, 39% improved). Although causal inferences cannot be drawn given the study design, these results suggest the therapeutic potential of Affinity in young people at elevated risk of suicide is worthy of further exploration.

Despite variability across participants, on average the Affinity system was well-used with participants on average logging almost five times per week, completing nearly 30 Steps, and posting or commenting almost 30 times in the Café. Completion of the therapy content is considered particularly high when compared to system usage in comparable online studies; for example, when the MOST platform was implemented for a similar time period with young people in remission from depression, the average number of Steps completed was 4.5 [32]. Exploratory non-parametric correlations also revealed a relationship between the number of Steps completed and improvements in depression symptoms and perceived burdensomeness. Additionally, relationships were found between several aspects of Affinity usage and higher ratings of Affinity’s safety, helpfulness, and ability to help control mood and suicidal thoughts. Again, although causality cannot be determined, these results are promising and worthy of further exploration.

Although overall Affinity was found to be safe, it is recognised that two participants made serious suicide attempts during the study and a small number deteriorated on self-reported clinical outcomes. Young people with complex depression at high risk of suicide were deliberately targeted as participants in this study, with more than half reporting a prior hospitalisation due to suicide risk and nearly all reporting clinically significant suicidal ideation scores at baseline. The occurrence of suicide attempts or deterioration in clinical status in at least some participants during the study period was therefore probable and expected [54]. Some researchers have in fact raised concerns about equating suicide attempts with adverse events in high-risk samples, arguing that such events are to be expected just as deaths by cancer are to be expected in trials of cancer treatment [55]. In the present study, all suicide attempts were considered adverse events; however, Affinity’s causative role in each was carefully evaluated by an experienced consultant psychiatrist and found to be absent. The lack of a causative relationship between Affinity use and suicide attempts (as opposed to the overall absence of adverse events) is therefore considered indicative of Affinity’s safety.

Despite the overall positive findings, there was less consensus regarding Affinity’s ability to help participants to feel more in control of their suicidal thoughts (mean rating of 3.2 out of 5). This should be interpreted in the context of the absence of therapeutic content overtly targeting cognitions related to suicide, and evidence that suggests interventions must directly target suicidal thoughts and behaviours if they are to effectively reduce these outcomes [56]. This was outside of scope for this initial pilot, as the primary objective was to confirm the safety and feasibility of the MOST model in this sample prior to investing in dedicated suicide related content. Additionally, the topic of suicidal thoughts or behaviours was not suggested as a Talk It Out topic, nor was it raised by any participant or moderator in the Café; in fact, participants were informed that posts related to suicide would likely be blocked by the system. Future iterations of Affinity will likely incorporate suicide-specific content and explore ways of allowing participants to discuss their experience of suicidal thoughts, should they want to do this.

A key strength of the Affinity platform relates to its safety features, including frequent moderation, keyword detection and blocking software, and prohibition of users communicating with each other outside the system. Despite allowing the trial to progress with no major safety concerns, it is acknowledged that these measures may have had unintended negative impacts. For example, although Affinity is designed to foster a sense of connectedness and belonging, the superficial and time-limited nature of the social network may have hindered participants’ ability or motivation to engage with the network and attempt to make meaningful connections. It is also possible that participants experienced the safety procedures as overly sensitive and deliberately censored themselves in an effort to avoid triggering a response from moderators; this has been identified as an issue associated with online interventions for suicide prevention more generally [20]. Moderators may also have experienced their role as particularly difficult or onerous; if so, this has implications for the feasibility and acceptability of Affinity. Forthcoming research from our team will report on qualitative data from both participants and moderators in an attempt to shed light on these issues.

### Limitations

In interpreting the findings of this study, several limitations should be considered. Firstly, the exploratory, uncontrolled nature of the study, small sample size, and concomitant treatment provided to all participants precludes any causative inferences from being made. Similarly, we were not able to examine mechanisms of action, such as whether change in interpersonal or cognitive targets, or both, moderated or mediated the relationship between platform use and improvement in depression or suicidal ideation; this will be a focus of future research. Secondly, due to the small sample size and exploratory nature of the correlations conducted, multiple comparisons were not adjusted for. It is acknowledged that this may increase the likelihood of Type 1 error and therefore these results should be interpreted with caution. Third, this study was conducted in a single specialised outpatient clinic and it is not known whether findings extend beyond this setting. Relatedly, it is not known whether findings would generalise to a significantly larger user base, or to users who are not engaged with mental health services; scaling the Affinity platform would likely require implementation of additional safety measures. Fourth, the study utilised a brief intervention period and participants were assessed at only two points, meaning no conclusions can be drawn regarding the long term impact of Affinity. Despite these limitations, however, this study provides vital evidence regarding the safety, feasibility, acceptability and potential therapeutic value of Affinity as an adjunct to face-to-face treatment, and will pave the way for large-scale controlled studies using this intervention.

## 5. Conclusions

Although historically concerns have been expressed about the safety of online social networking interventions for people who experience suicidal ideation, particularly regarding their potential to lead to contagion of suicidal ideation and/or behaviour, the results of this study provide world-first evidence to suggest that such interventions can be implemented safely when appropriate moderation and risk management protocols are in place. Affinity was not only found to be safe, but also highly acceptable and feasible to implement. This study suggests that future research projects with the MOST software should not exclude participants based on high suicide risk. Furthermore, although the results of this study need to be replicated via a larger randomised controlled trial, they have significant implications for the future of supporting young people who experience suicidal ideation given the potential for online peer-to-peer social networking to target interpersonal risk factors for suicide (perceived burdensomeness and thwarted belongingness) [15]. Researchers who design and evaluate internet-based interventions for people at risk of suicide should, in addition to delivering evidence- and theory-based therapeutic content, consider harnessing the benefits afforded by the internet by allowing for peer-to-peer social networking.

## Figures and Tables

**Table 1:** Demographic characteristics of the sample.

Characteristics	*N* (%)
**Age (M (SD))**	21.7 (2.7)
**Gender**	
Male	7 (35)
Female	11 (55)
Transgender	2 (10)
**Marital status**	
Married/de facto	1 (5)
In a relationship	3 (15)
Single	16 (80)
**Sexual orientation**	
Heterosexual	12 (60)
Gay	1 (5)
Bisexual	3 (15)
Questioning	3 (15)
Other	1 (5)
**Country of birth**	
Australia	15 (75)
Asia	4 (20)
United Kingdom	1 (5)
**Aboriginal or Torres Strait Islander**	
Yes	0
No	20 (100)
**Highest level of education completed**	
High school	5 (25)
Certificate or diploma	4 (20)
Bachelor degree	5 (25)
Postgraduate degree	2 (10)
**Currently studying or training**	
Yes	13 (65)
No	6 (30)
**Currently employed**	
Yes	12 (60)
No	8 (40)

**Table 2:** Clinical characteristics of the sample at baseline.

Characteristics	*N* (%)
**Planning or attempting suicide**	
Ever planned suicide	8 (40)
Ever attempted suicide	11 (55)
Planned or attempted suicide within the past six months	12 (60)
Ever been hospitalised for psychological or psychiatric reasons	13 (65)
**Self-harm**	
Ever self-harmed	16 (80)
…Self-harmed by cutting	13 (81)
…Self-harmed by burning with cigarette, lighter or match	5 (31)
…Self-harmed by carving words, pictures or designs into skin	6 (38)
…Self-harmed by scratching	10 (63)
…Self-harmed by biting	2 (13)
…Self-harmed by sticking or rubbing sharp objects into skin	5 (31)
…Self-harmed by banging head or punching self	10 (63)
**PHQ-9 score**	
Minimal or none	0
Mild	0
Moderate	2 (10)
Moderately severe	7 (35)
Severe	10 (50)
**ASIQ score**	
<31	2 (10)
≥31 (clinical range)	18 (90)

ASIQ = Adult Suicidal Ideation Questionnaire; PHQ-9 = Patient Health Questionnaire-9.

**Table 3:** Means (M), standard deviations (S.D.), and within-group effect sizes (Cohen’s d) for therapeutic targets and clinical outcomes.

Variable	Baseline		8-Week Follow-Up		*p*	d	RCI (N, %)		N
	M	SD	M	SD			Improve	Decline	
**Clinical outcomes**									
Suicidal Ideation (ASIQ)	91.5	36.8	75.5	36.6	0.033	−0.57	7, 41%	2, 12%	17
Depression (PHQ-9)	19.6	4.1	15.5	6.9	0.016	−0.94	7, 41%	1, 6%	17
**Interpersonal targets**									
Perceived Burdensomeness (INQ-PB)	26.7	10.3	21.6	11.1	0.048	−0.52	7, 39%	3, 17%	18
Thwarted Belongingness (INQ-TB)	44.1	10.0	35.2	14.4	0.006	−0.96	10, 56%	3, 17%	18
Social Connectedness (SCS)	21.6	9.5	27.9	12.2	0.062	0.54	8, 44%	2, 11%	18
**Cognitive targets**									
Mindfulness (MAAS)	2.9	0.6	3.1	0.8	0.411	0.38	2, 11%	4, 22%	18
Self-Compassion (SCS-SF)	1.8	0.6	2.2	0.6	0.004	0.82	10, 56%	0, 0%	18
Problem Solving (NPOQ)	42.9	8.8	41.1	11.2	0.286	−0.30	8, 44%	5, 28%	18

ASIQ = Adult Suicidal Ideation Questionnaire; PHQ-9 = Patient Health Questionnaire-9; INQ-PB = Interpersonal Needs Questionnaire – Perceived Burdensomeness; INQ-TB = Interpersonal Needs Questionnaire – Thwarted Belongingness; SCS = Social Connectedness Scale; MAAS = Mindful Attention Awareness Scale; SCS-SF = Self-Compassion Scale-Short Form; NPOQ = Negative Problem Orientation Questionnaire.

**Table 4:** Participant impressions of the Affinity intervention.

Item	M	SD	Mdn	Range	N (%) Rating ≥ 3
How positive was your experience on Affinity? (N = 17)	4.6	0.51	5	4–5	17 (100)
How helpful did you find Affinity? (N = 17)	4.4	0.79	5	3–5	17 (100)
How easy was it to use Affinity? (N = 17)	4.2	0.75	4	3–5	17 (100)
How safe did you feel on Affinity? (N = 17)	4.8	0.56	5	3–5	17 (100)
How much has using Affinity helped you feel more in control of your mood? (N = 16)	3.8	0.91	4	2–5	15 (94)
How much has using Affinity helped you feel more in control of suicidal thoughts? (N = 16)	3.2	1.05	4	1–5	13 (81)

**Table 5:** Activity on Affinity.

Variable	Range	M	SD	Mdn
Total logins	1–754	78.7	163.1	38.5
Weekly logins	0–33	4.7	7.4	2.3
Café posts and comments	1–177	28.7	49.7	7.5
Café replies received	0–165	30.7	47.7	11.5
Talk It Out posts and comments	0–20	2.5	5.4	0
Steps completed	1–120	29.2	37.5	11.5
Actions completed	0–68	13.7	21.7	4.5
Likes and reactions given	0–192	37.1	55.8	6.0
Likes and reactions received	6–347	69.7	100.8	31.0
SMSs received from moderator	2–14	6.9	3.2	7.0
SMSs sent to moderator	0–4	1.2	1.0	1.0

**Table 6:** Exploratory non-parametric correlations between change in clinical outcomes, subjective ratings of Affinity, and key domains of Affinity use.

Variable	Total Logins	Weekly Logins	Café Posts and Comments	Steps Completed	Do−Its Done	Talk It Out Posts/Comments	Likes and Reactions Made	Likes and Reactions Received	SMSs Received from Moderator	SMSs Sent to Moderator
ASIQ	−0.19	−0.31	−0.14	−0.47	−0.2	−0.32	−0.1	−0.2	0.16	−0.3
PHQ−9	−0.28	−0.39	−0.11	−0.61 **	−0.32	−0.48	−0.09	−0.16	0.44	−0.08
INQ−PB	−0.3	−0.42	−0.14	−0.60 **	−0.4	−0.31	−0.07	−0.21	0.38	−0.36
INQ−TB	−0.17	−0.25	−0.15	−0.27	−0.23	−0.17	0.01	−0.17	0.13	−0.53 *
SCS	0.07	0.23	0.01	0.14	0.11	−0.2	−0.08	−0.04	−0.19	0.47 *
MAAS	−0.15	−0.15	−0.19	0.08	−0.05	−0.05	−0.09	−0.23	−0.24	−0.08
SCS−SF	0.1	0.16	0.17	0.28	0.26	0.23	0.09	0.21	−0.33	0.3
NPOQ	−0.22	−0.1	−0.09	−0.24	−0.01	−0.46	0.04	−0.12	0.13	0.31
SR: Positive	0.33	0.19	0.32	0.29	0.31	0.31	0.26	0.35	−0.22	0.03
SR: Helpful	0.34	0.28	0.47 *	0.39 *	0.46 *	0.34	0.56 **	0.54 **	−0.18	0.11
SR: Easy	0.12	0.26	−0.02	0.33	0.23	0.08	0.11	0.17	0.07	0.09
SR: Safe	0.11	0.07	0.06	0.31	0.14	0.13	0.03	0.16	−0.07	0.44 *
SR: Improve Mood	0.45 *	0.37	0.56 **	0.36	0.53 **	0.31	0.60 **	0.54 **	−0.19	0.12
SR: Reduce SI	0.31	0.18	0.48 *	0.09	0.10	0.23	0.42 *	0.34	−0.07	0.04

ASIQ = Adult Suicidal Ideation Questionnaire; PHQ-9 = Patient Health Questionnaire-9; INQ-PB = Interpersonal Needs Questionnaire – Perceived Burdensomeness; INQ-TB = Interpersonal Needs Questionnaire – Thwarted Belongingness; SCS = Social Connectedness Scale; MAAS = Mindful Attention Awareness Scale; NPOQ = Negative Problem Orientation Questionnaire; SCS-SF = Self-Compassion Scale-Short Form; SR = Subjective Rating of Affinity. *. Correlation is significant at the 0.05 level (2-tailed). **. Correlation is significant at the 0.01 level (2-tailed).
